# New Roles of the Primary Cilium in Autophagy

**DOI:** 10.1155/2017/4367019

**Published:** 2017-08-23

**Authors:** Yenniffer Ávalos, Daniel Peña-Oyarzun, Mauricio Budini, Eugenia Morselli, Alfredo Criollo

**Affiliations:** ^1^Departamento de Fisiología, Facultad Ciencias Biológicas, Pontificia Universidad Católica de Chile, 7820436 Santiago, Chile; ^2^Advanced Center for Chronic Diseases (ACCDiS), Facultad Ciencias Químicas y Farmacéuticas & Facultad de Medicina, 8380494 Santiago, Chile; ^3^Center for Molecular Studies of the Cell (CEMC), Facultad de Medicina, 8380453 Santiago, Chile; ^4^Instituto de Investigación en Ciencias Odontológicas (ICOD), Facultad de Odontología, 8380492 Santiago, Chile

## Abstract

The primary cilium is a nonmotile organelle that emanates from the surface of multiple cell types and receives signals from the environment to regulate intracellular signaling pathways. The presence of cilia, as well as their length, is important for proper cell function; shortened, elongated, or absent cilia are associated with pathological conditions. Interestingly, it has recently been shown that the molecular machinery involved in autophagy, the process of recycling of intracellular material to maintain cellular and tissue homeostasis, participates in ciliogenesis. Cilium-dependent signaling is necessary for autophagosome formation and, conversely, autophagy regulates both ciliogenesis and cilium length by degrading specific ciliary proteins. Here, we will discuss the relationship that exists between the two processes at the cellular and molecular level, highlighting what is known about the effects of ciliary dysfunction in the control of energy homeostasis in some ciliopathies.

## 1. Introduction

The primary cilium (PC) is a microtubule-based antenna-like structure that emanates from the surface of different cell types [1]. Primary cilia receive signals from the environment and control different intracellular signaling pathways [1]. Importantly, it has become clear that the PC contributes to metabolic regulation; indeed, lacking the PC, as well as altered cilia length, dysregulates energy balance and promotes obesity [2, 3]. Here, we will summarize studies that describe the role of the PC in the regulation of energy homeostasis.

Another cellular process required to maintain cellular and tissue homeostasis is macroautophagy, hereafter referred to as autophagy. This process occurs in all cell types and is constitutively active, allowing the removal of old and potentially toxic proteins and organelles to preserve cellular function [4]. Recently, a bidirectional interplay between autophagy and the PC has been demonstrated: cilium-dependent signaling is necessary for autophagosome formation and, conversely, autophagy regulates both ciliogenesis and cilium length by degrading specific ciliary proteins [5, 6]. In addition to the role of the PC in energy homeostasis, we will review studies focusing on the crosstalk between these two processes at the molecular and cellular level, highlighting any physiological and pathological implications.

## 2. The Primary Cilium: Structure and Functions

As previously mentioned, the PC is a nonmotile antenna-like structure formed by the process of ciliogenesis [7]. The PC emanates from the surface of almost all cell types, depending on cell cycle and differentiation stage, and acts as a sensor of different extracellular signals such as growth factors, hormones, light, mechanical stimuli, odorants, and developmental morphogens, as reviewed elsewhere [1, 8, 9].

**Figure 1:**
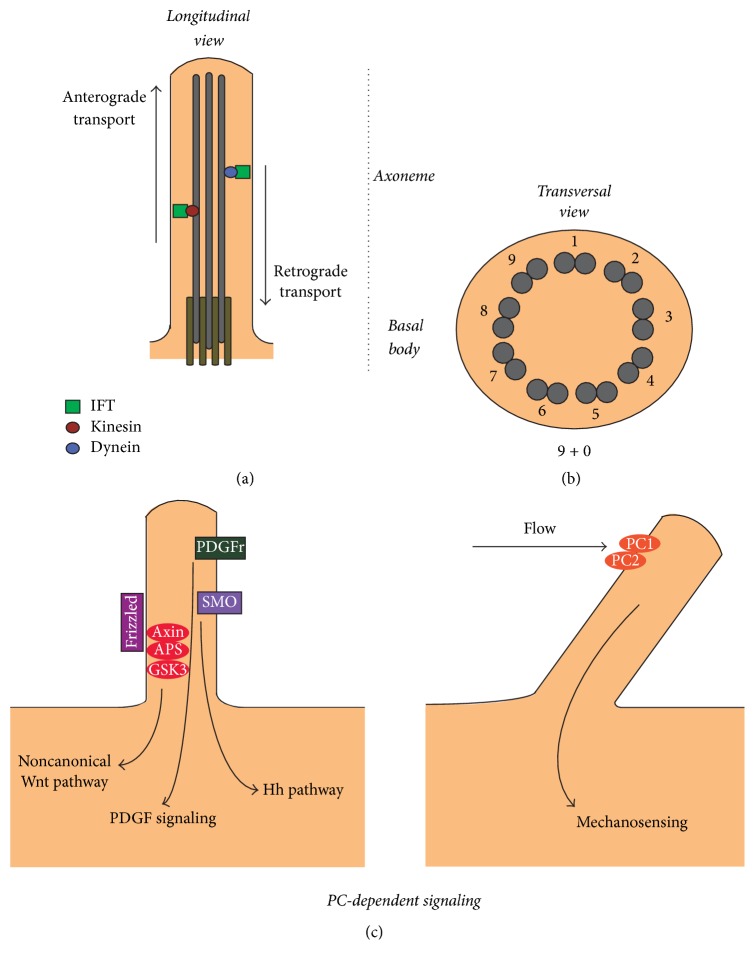
Primary cilium: structure and function. (a) Longitudinal representation of the primary cilium shows an axoneme center formed by highly stable acetylated microtubules that arise from the basal body parked near the nucleus. The components that are transported to the primary cilium (anterograde transport) rely on intraflagellar transport (IFT) proteins attached to Kinesins motor proteins. Conversely, transport from the primary cilium (retrograde transport) depends on different IFT proteins and dynein motor proteins. (b) Transversal representation of the primary cilium shows a 9 + 0 microtubule array, explaining the nonmotile behavior of the primary cilium. (c) Signaling of the primary cilium. On the left, several receptors present at the primary cilium such as platelet-derived growth factor receptor (PDGFR) for activation of the ERK pathway and Patched-1 (PTCH1) activated by Hh ligand for smoothened (SMO) translocation to the ciliary tip and activation of the transcription factor glioma (GLI). On the right, flow activates mechanosensation pathways by activation of the PC1/PC2 calcium channel and noncanonical activation of the Wnt pathway by increasing the expression of inversin in a calcium dependent mechanism, which participates in the degradation of APC and accumulation of *β*-catenin.

The PC itself is composed of a microtubule-based core structure named the axoneme, which nucleates from a basal body and is surrounded by a ciliary membrane, which is continuous with the plasmatic membrane (Figure 1). The ciliary membrane is enriched in different receptors; thus, the PC represents a signaling platform [10–12].

The axoneme of the PC comprises nine parallel doublets of microtubules, which form a ring. This configuration is known as “9 + 0” and is seen in all nonmotile cilia. In contrast, motile cilia have an extra central pair of microtubules, arranged in what is known as “9 + 2” structure [7]. The tubulin is subjected to multiple posttranslational modifications, including acetylation, glutamylation, and glycylation, that regulate the assembly and the maintenance of the ciliary structure [13].

Briefly, the process of PC assembly begins with the formation of the basal body, the mother centriole from which the cilium nucleates, and its migration and docking to the actin-rich cortex located at the cell surface. During this process, different membrane vesicles associate with the basal body and, after the fusion with the plasma membrane, constitute the ciliary membrane. Subsequently, the axonemal microtubules elongate from the distal end of the outer doublets and the cilium arises [14]. The process of PC assembly has been revised by Sanchez and Dynlacht [15].

The PC is highly compartmentalized and lacks the ability to synthesize protein; thus, it requires a transport process to transfer and correctly localize the proteins necessary for the growth, maintenance, and signaling of the PC. This process is carried out by the intraflagellar transport (IFT) which is a bidirectional transport system mediated by multiprotein complexes (known as IFT particles) along the axoneme [16, 17]. The transport of ciliary proteins from the cytoplasm to the ciliary tip, known as anterograde transport, depends on the heterotrimeric kinesin-2 protein, which consists of two motor subunits, KIF3A and KIF3B, and an accessory subunit, the kinesin-associated protein (KAP) [18]. Likewise, the IFT particle involved in the anterograde transport is the IFT complex B (IFT-B), which comprises 15 proteins (IFT20, IFT22, IFT25, IFT27, IFT46, IFT52, IFT54, IFT57, IFT70, IFT74, IFT80, IFT81, IFT88, and IFT172) [19]. Interestingly, both the kinesin-2 and IFT-B complexes participate in the anterograde transport and are necessary for the assembly and maintenance of the PC [17]. Consequently, the knockdown of components of these complexes results in short or absent PC [2, 20–22].

On the other hand, the transport from the ciliary tip to the cytoplasm is mediated by the cytoplasmic dynein 2 complex and by the IFT complex A (IFT-A). The IFT-A comprises six proteins (IFT43, IFT121, IFT122, IFT139, IFT140, and IFT144) and is required for the retrograde transport [16]. Depletion of components of the retrograde transport system produces short and swollen cilia and leads to the accumulation of different proteins in the PC [23].

The importance of the PC is highlighted by the presence of a group of diseases known as ciliopathies, which are characterized by disruption of the PC. Loss of the PC causes cystic kidney disease, neurodevelopmental anomalies, blindness, obesity, polydactyly, and, sometimes, insulin resistance and type 2 diabetes. Human ciliopathies have been recently revised elsewhere [1, 24]. Pathological conditions also include polycystic kidney disease (PKD), the Joubert syndrome, the Meckel-Grüber syndrome (MKS), the oral-facial-digital syndrome (OFD), the Alström syndrome (ALMS), and the Bardet-Biedl syndrome (BBS) (reviewed by [24, 25]). Importantly, BBS and ALMS are characterized by being metabolic disorders.

The BBS is a syndromic ciliopathy characterized by cystic kidney disease, obesity, retinitis pigmentosa, hypogonadism, polydactyly, and intellectual and behavioral disabilities [26]. BBS is genetically heterogeneous and is caused by different mutations in BBS genes. The BBS proteins localize in the ciliary basal body and axoneme, and many of them are components of the so-called BBSome. The BBSome is a stable protein complex involved in the biogenesis of the PC, which is formed by seven highly conserved BBS proteins (BBS1, BBS2, BBS4, BBS5, BBS7, BBS8, and BBS9) and by the protein BBSome-interacting protein of 10 KDa (BBIP10) [27–29].

The ALSM is a rare ciliopathy characterized by obesity, progressive vision and hearing loss, hyperinsulinemia, type 2 diabetes, and cardiomyopathy [30, 31]. ALSM is caused by mutations in the* Alsms1* gene, which codes for the ALSM1 protein expressed in most tissues, whose function is unknown. The syndrome is caused by loss of function mutations that generate a short and nonfunctional version of the protein.

As previously mentioned, the existence of a functional interaction between the PC and autophagy has recently been demonstrated. Below, we will briefly introduce the process of autophagy and its mechanisms of regulation and we will review the role of the PC in the development of ciliopathies, specifically in the context of the crosstalk between autophagy and PC.

## 3. Mechanism of Autophagy Regulation

Autophagy (from the Greek “self-eating”) is a bulk degradation pathway highly conserved from yeast to mammals, in which components that will be recycled are sequestered into double-membrane vesicles, known as autophagosomes. Autophagosomes fuse with lysosomes, vesicle-shaped organelles with hydrolytic enzymes in their luminal region, thus allowing the degradation of the material enclosed into the autophagosomes [39].

Autophagy has been reported to have a dual role being both cell-protective and cell-destructive. “Autophagic cell death,” also known as type 2 cell death, is morphologically defined as a type of cell death that occurs without chromatin condensation and in presence of autophagic vacuoles in the cytoplasm. However, an accumulating body of literature identifies autophagy as a cell survival/cytoprotective process in the vast majority of pathophysiological and experimental settings, as autophagy inhibition generally accelerates the death of cells experiencing altered homeostasis [40, 41]. Indeed, cells use autophagy to maintain their physiological homeostasis, that is, for protein degradation, organelle recycling, and energy generation. Nevertheless, autophagy is also an inducible stress mechanism and increases in response to nutrient deprivation, bacterial infections, and protein misfolding to promote cell survival [42, 43]. Because of the critical role of autophagy as an adaptive process, its dysregulation provokes pathologies such as cancer, neurodegeneration, and metabolic disorders [44–46]. The role of autophagy in the onset of human diseases has been revised in Schneider and Cuervo [47].

**Figure 2:**
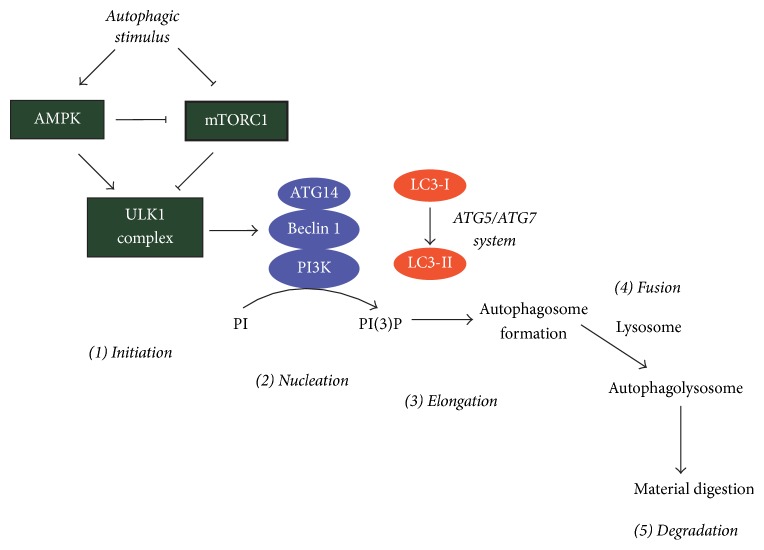
Overview of the autophagic pathway. Autophagy can be divided into five stages: initiation, nucleation, elongation, fusion, and degradation. “Initiation” refers to the activation of the ULK1 (ATG1) complex, which can be achieved by increased activity of AMP-dependent kinase (AMPK) and/or inhibition of the mechanistic target of rapamycin complex 1 (mTORC1). The “nucleation” indicates the process of recruitment of proteins of the autophagic machinery at the autophagosome formation sites, which are required for the formation of the new autophagosome, which occurs during the “elongation” stage. Then the complete autophagosome is loaded with the material that needs to be removed from the cell and degraded. The autophagosome fuses with a lysosome, thus activating the lysosomal acidic hydrolases, which degrade the cargo sequestered in the autophagic vesicle. See the main text for additional details.

Autophagy is a process performed by “autophagy-related proteins” (ATGs) and it can be divided into five stages: initiation, nucleation, elongation, fusion, and degradation [48] (Figure 2). In brief, “initiation” refers to the activation of the ULK1 (ATG1) complex, which can be achieved by increased activity of AMP-dependent kinase (AMPK) and/or inhibition of the mechanistic target of rapamycin complex 1 (mTORC1). In basal conditions, cells have a controlled level of autophagy; mTORC1 phosphorylates ULK1 at ser-737, thereby suppressing its activity. However, autophagic stimuli (i.e., nutrient deprivation) activate AMPK, which both inhibits mTORC1 and phosphorylates ULK1 at ser-317 and ser-777. Unlike mTORC1, the phosphorylation of ULK1 by AMPK leads to its activation [49]. The “nucleation” stage starts when the active ULK1 complex translocates to autophagosome formation sites. These sites include endoplasmic reticulum-mitochondrial contact sites, the plasmatic membrane, or the PC itself [5, 50, 51]. Subsequent to translocation, ULK1 phosphorylates Beclin 1 (ser-14), which, together with Vps34 and Vps15, forms class III phosphatidylinositol 3-kinase (PI3K) complex [52]. The phosphorylation of Beclin 1 provokes a conformational change that allows its interaction with several coactivators (i.e., ATG14, UVRAG), increasing the catalytic activity of the phosphatidylinositol-4,5-bisphosphate 3-kinase (PI3K) complex [52]. Once active, the PI3K complex promotes the synthesis of phosphatidylinositol 3-phosphate to recruit FYVE- and PX-domain containing ATGs that participate in the formation of the autophagosome [50]. During “elongation,” the preautophagic membranes expand, ultimately forming the autophagosome. The microtubule-associated protein 1A/1B-light chain 3 (MAP1LC3, or just LC3) is a protein normally found in the cytoplasm (LC3-I), but, in autophagic conditions, a phosphatidylethanolamine is covalently attached by ATG3 (LC3-II), allowing the LC3 protein to relocate and form autophagosomes [53]. Given that LC3 is part of the autophagosome, LC3 is considered a classic autophagic marker. The incorporation of LC3 into autophagosomes is also mediated by the ATG5/ATG12 complex, which, together with ATG16L, forms a new complex that functions similarly to an E3 enzyme [54]. When the autophagosome structure is complete and the vesicle is loaded with the material that needs to be degraded, the autophagosome fuses with a lysosome, a vesicle-shaped organelle that contains a low luminal pH and a battery of hydrolytic enzymes, promoting the degradation of the material sequestered by the autophagosome [55]. A classic marker of autophagic degradation is p62/SQSTM1, a protein that recognizes poly-ubiquitinated proteins destined for recycling, and attaches to autophagosomes through a LC3-interacting region (LIR). Thus, when autophagy increases, p62/SQSTM1 levels decrease [56]. The protein p62/SQSTM1 is used as marker of the “autophagic flux,” a term that describes the lifecycle of autophagosomes from synthesis to degradation within the lysosome. The study of the autophagic flux is required to understand if the increase in autophagy is an “on-rate” process, in which new autophagosomes are continually formed, or an “off-rate” process, in which the already formed autophagosomes are not degraded by the lysosomes [57].

The detailed mechanisms of autophagy regulation have been recently revised elsewhere [58].

As previously mentioned, the PC has been identified as a site of formation of autophagosomes, and cilium-dependent signaling is necessary for autophagosomes formation [59]. Interestingly, the interplay between autophagy and PC seems bidirectional, since additional studies indicate that autophagy regulates ciliogenesis by degrading specific ciliary proteins [6, 60]. The crosstalk between autophagy and the PC will be discussed in the next section.

## 4. Autophagy and Primary Cilium Crosstalk

**Figure 3:**
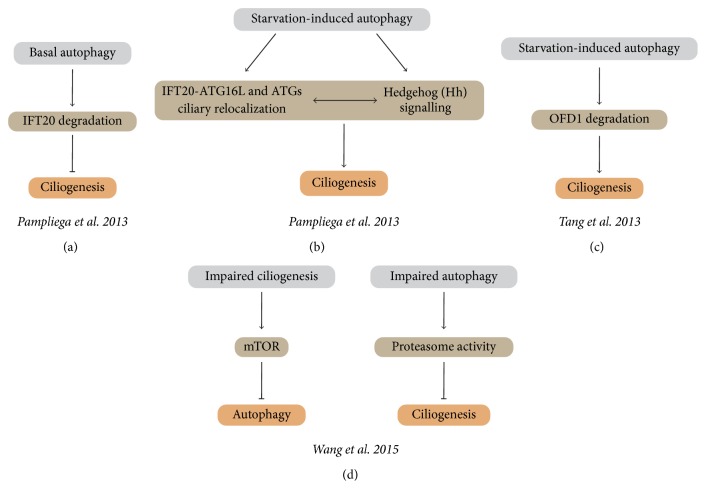
Autophagy and primary cilium crosstalk. Summary of the published studies indicating the existence of a bidirectional crosstalk between ciliogenesis and the primary cilium. (a-b) Pampliega et al. show that proteins of the autophagic machinery localize at the primary cilium. In basal conditions, autophagy degrades the ciliary protein intraflagellar transport 20 (IFT20), inhibiting ciliary growth. In addition, when autophagy is induced by serum starvation, ciliogenesis is promoted by a cilium-dependent activation of the Hedgehog pathway. (c) Tang et al. show that ciliogenesis is induced in conditions of serum starvation through autophagy-mediated degradation of the protein oral-facial-digital syndrome 1 (OFD1). (d) Wang et al. work indicates that autophagy and primary cilium reciprocally affect each other: inhibition of ciliogenesis is associated with the activation of mechanistic target of rapamycin (mTOR) and consequently with the inhibition of autophagy. On the other side, a condition of impaired autophagy stimulates the proteasome, which inhibits ciliogenesis. See the main text for additional details.

The first studies showing an interplay between autophagy and ciliogenesis are the works by Tang et al. and by Pampliega et al., which were copublished in 2013 [5, 6] (Figures 3(a)–3(c)). Tang and collaborators show that the centriolar satellite protein oral-facial-digital syndrome 1 (OFD1) coimmunoprecipitates with LC3. Interestingly, the levels of OFD1 are reduced in wild-type (WT) mouse embryonic fibroblasts (MEFs) subjected to serum starvation, an effect that is abrogated in autophagy-deficient Atg5^−/−^ MEFs. Likewise, the pharmacological inhibition of the autophagic flux, using bafilomycin A1 (BafA1) or chloroquine (CQ), results in OFD1 accumulation in serum-starved cells. Altogether, these results suggest that OFD1 is degraded by serum deprivation-induced autophagy [6]. Additionally, they show that autophagy also regulates the percentage of ciliated cells and the length of this organelle during starvation. Briefly, ciliogenesis induced by serum deprivation is partially inhibited in Atg5^−/−^ and Atg3^−/−^ MEFs. Indeed, these cells show a lower percentage of ciliated cells and diminished cilia length. Likewise, the treatment of WT MEFs with the late autophagy inhibitor CQ produces the same effects [6]. Importantly, the knockdown of* Ofd1* in autophagy-deficient cells restores the serum deprivation-induced ciliogenesis and cilia length, suggesting that the autophagic degradation of OFD1 is necessary for starvation-induced ciliogenesis. Interestingly, the knockdown of* Ofd1* also increases cilia formation in basal conditions. To confirm the role of OFD1 in the regulation of ciliogenesis, the authors knocked down* Ofd1* in MCF7 cells which lack PC and observed an increase in the percentage of cells with cilia after serum withdrawal [6]. Altogether, these results suggest that OFD1 is a negative regulator of ciliogenesis in basal conditions, but its degradation by serum starvation-induced autophagy is sufficient and necessary to promote ciliogenesis in different cell types.

The close interaction between PC-dependent signaling pathways, ciliogenesis, and autophagy was confirmed by the study of Pampliega et al. [5] (Figures 3(a) and 3(b)). The authors used two cellular models of impaired ciliogenesis: the stable knockdown of IFT20 in MEFs (IFT20(−)) and kidney epithelial cells (KECs) from mice with a hypomorphic mutant of IFT88 (IFT88^−/−^). Both IFT20 and IFT88 are parts of the IFT-B complex, which is essential for ciliogenesis [22]. In both models, PC formation is significantly reduced in conditions of serum starvation. Importantly, in the same models and in the same experimental conditions that reduced PC formation, the autophagic flux is also significantly inhibited [5]. These results suggest that compromised ciliogenesis impairs autophagy induction following serum withdrawal [5]. The authors further elucidated the mechanism involved and evaluated the role of the Hedgehog (Hh) signaling pathway, which is a PC-dependent pathway that requires an intact IFT, in autophagy regulation. First, they show that the pharmacological or genetic activation of the Hh pathway induces autophagy in MEFs. However, the treatment with an activator of the Hh pathway (Purmorphamine) does not increase the autophagic flux in IFT20(−) MEFs and IFT88^−/−^ KECs when compared to WT MEFs, indicating that an intact IFT is required for the proper activation of the Hh signaling pathway. To confirm these results, Pampliega et al. overexpressed GLI1, an effector of the Hh pathway, in IFT88^−/−^ KECs, and this reversed the lack of starvation-induced autophagy. Additionally, reduction of Hh signaling decreased starvation-induced autophagy in the same cellular model. Altogether, these results indicate that the Hh signaling pathway is necessary for the upregulation of PC-dependent autophagy following serum withdrawal [5].

The work of Pampliega et al. also demonstrates that some autophagy-related proteins associate with different ciliary structures in an IFT and serum dependent manner. ATG16L relocalizes at the basal body of the PC in conditions of serum starvation or when ciliary Hh signaling is increased. ATG16L colocalizes and coimmunoprecipitates with IFT20, which allows its trafficking from the Golgi to the base of the PC, in conditions of serum withdrawal [5]. The authors propose that the protein IFT20 represents the mechanism through which autophagy regulates PC growth, suggesting that IFT20 is degraded by basal autophagy, thus maintaining ciliogenesis at a minimum level. To support this hypothesis, Pampliega et al. showed that IFT20 protein accumulates in autophagy-deficient Atg5^−/−^ MEFs and promotes PC growth. Indeed, Atg5^−/−^ MEFs cultured in normal medium or in starvation conditions exhibit longer cilia and a higher percentage of ciliated cells. Likewise, the knockdown of other* Atgs* or pharmacological inhibition of autophagy induces ciliogenesis in different cell types, suggesting that stimulation of autophagy reduces ciliogenesis.

The study of Pampliega et al. regarding the role of basal autophagy in ciliogenesis conflicts with the results obtained by Tang et al. (2013) [6]; however, the authors suggest that the differences could be explained by the state of confluence of the cells [61].

In agreement with the importance of IFT20 in the crosstalk between the autophagic machinery and ciliogenesis, it has recently been demonstrated that humans carrying a mutation in the gene* Vps15* show some features characteristic of ciliopathies, such as retinitis pigmentosa, renal dysfunction, and developmental anomalies [62]. Interestingly, skin fibroblasts derived from these patients show shorter PC; however, no differences were found in the interaction between the mutated form of VPS15 (VPS15-R998Q) and VPS34, UVRAG, Beclin 1, or Atg14L, which, as previously indicated, are required in the early steps of autophagosomes formation [62]. Stoetzel et al. identified a difference in the localization of the protein IFT20 between normal and skin fibroblasts from patients when cells are subjected to serum deprivation. In fibroblasts derived from patients, IFT20 localized only to the Golgi, while in normal fibroblasts IFT20 was found in the cytoplasm, the PC, and the Golgi [62]. This suggests that mutation of VPS15 alters IFT20-dependent Golgi to PC trafficking, thus impairing ciliogenesis. Importantly, the effect of VPS15 is independent of its interaction with VPS34, suggesting that autophagy is not involved in this mechanism [62]. More recently, consistently with the work of Pampliega et al. and Tang et al. [5, 6], an additional study confirms that autophagy and the PC reciprocally affect each other [60] (Figure 3(d)). Interestingly, in addition to the previous works, Wang and colleagues show that the crosstalk between PC and autophagy involves the mTOR pathway, whose suppression promotes autophagy and ciliogenesis, and the proteasomal degradation pathway, whose autophagy-mediated inhibition stimulates ciliogenesis [60]. Wang et al. used IFT88-KD2 cells (HK2 cells in which IFT88 was knocked down) and C13 cells (selected from kidney epithelial cells with short cilia) to demonstrate that basal and starvation-induced autophagy is reduced in both types of cilia-deficient cells. Interestingly, these cells showed higher activation of the mTOR pathway, as indicated by the phosphorylation status of its downstream substrates, and treatment with rapamycin restored autophagy in both cell types. These results suggest that PC depletion inhibits autophagy through the activation of the mTOR signaling pathway [60]. Additionally, autophagy induction in IFT88-KD2 cells significantly increases PC length, while its inhibition shortens cilia in the same cellular model. Consistently, PC length and frequency are reduced in kidney proximal tubular cells from* Atg7*-knockout mice compared with WT mice. Interestingly, the inhibition of proteasomal activity with MG132 reverses the shortening of cilia in cells treated with the autophagy inhibitor 3-methyladenine (3-MA) and similar results were obtained in Atg5^−/−^ MEFs when compared with WT MEFs. Moreover, proteasome activity is higher in Atg5^−/−^ MEFs than WT cells, suggesting that the inhibition of autophagy promotes proteasomal degradation of an unknown protein that is necessary for ciliogenesis [60]. Altogether, these results indicate that a functional PC of normal length is required for basal and starvation-induced autophagy and, conversely, autophagy is necessary for ciliogenesis and for the maintenance of cilia length.

On the other hand, it is known that the PC acts as a flow sensor at the urinary level [63, 64] and regulates kidney epithelial cell size through the liver kinase B1- (LKB1-) AMPK-mTOR pathway [65]. Therefore, considering the crosstalk between PC and autophagy, Orhon et al. [64] evaluated whether autophagy is involved in the control of epithelial cell volume in response to fluid flow. The authors showed that increased fluid flow induces autophagy and decreases cell volume in Madin-Darby canine kidney (MDCK) epithelial cells. These results were confirmed in WT KECs; however, in KECs carrying a hypomorphic deletion of the Ift88 allele (IFT88^−/−^), which abrogates ciliogenesis, the induction of autophagy and the reduction of cell volume were prevented. The same results were also obtained in KECs following the knockdown of KIF3A when exposed to increased fluid flow, suggesting that the PC is required for fluid flow-induced autophagy [64]. Moreover, in KECs, in which autophagy is inhibited due to the knockdown of* Atg5* or* Atg16l*, modifications in the fluid flow do not affect cell volume, suggesting that flow-induced autophagy is required for the regulation of cell size. Interestingly, in agreement with the work of Pampliega et al., this flow-induced autophagy is characterized by the recruitment of ATG16L to the basal body of the PC, suggesting that ATG16L translocation might be the hallmark of PC-dependent autophagy. Importantly, the interruption of urinary flow in vivo, caused by unilateral ureteral obstruction (UUO), inhibits autophagy and increases tubular epithelial cell size in mice compared to sham-operated controls. Likewise, systemic inhibition of autophagy through CQ administration increases the size of epithelial cells in kidneys when compared to vehicle-treated mice [64].

Additionally, Orhon et al. evaluated the role of the PC in vivo in the regulation of autophagy and cell size in kidney epithelial cells. KIF3A-deficient mice, where KIF3A is selectively depleted in tubular cells, exhibited impaired ciliogenesis, reduced autophagy, and larger tubular epithelial cells than control mice. Altogether, these results indicate that PC-dependent flow-induced autophagy is required for the control of cell size in vivo [64]. These effects depend on the activation of the mTOR pathway, which is initially activated by changes in fluid flow. Interestingly, the knockdown of LKB1 reduced flow-induced autophagy and prevented the decrease in cell volume promoted by fluid flow in KECs, suggesting that the LKB1-AMPK-mTOR pathway is involved in fluid-flow-induced autophagy and cell volume regulation [64]. Altogether, these results indicate there is a close relationship between ciliogenesis, maintenance of cilia length, and autophagy both in vitro and in vivo. At the molecular level, both processes are connected by the Hh and the AMPK-mTOR signaling pathways and depend on the localization of autophagy-related proteins at the basal body and axoneme of the PC and through the control of transport of proteins relevant for ciliogenesis. However, many questions regarding the molecular mechanisms involved in the interplay between PC and autophagy remain unanswered. Additionally, are ciliopathies characterized by alteration in autophagy levels?

## 5. Polycystic Kidney Disease (PKD)

PKD is one of the most common ciliopathies, affecting 1 : 500–1 : 1000 people around the world. The disease is characterized by the presence of multiple growing cysts on the kidney that could ultimately lead to end-stage renal disease [66]. Several investigations suggest that cysts arise from a hyperactive mTORC1 signaling; indeed, immunohistochemistry of PKD patients shows increased levels of phospho-mTORC1 along with high levels of phospho-S6K protein, a downstream target of mTORC1 [67]. Furthermore, treatment with rapamycin, a chemical inhibitor of mTORC1, attenuates cyst growth in animal models of PKD [67] and patients [68]. In order to represent and understand what happens in humans, multiple animal models of PKD have been generated which induce cilia dysfunction at the kidney by mutating the cilia-associated protein CYSTIN in mice (cpk model) [69] or the protein SAMCYSTIN in rats (Han:SPRD model) [70].

In humans, PKD is mainly caused by mutations in the gene coding for Polycystin-1 (PC1). PC1 protein is enriched at the PC and functions to transduce signals from the environment to the cell [71]. Conditional reduction of PC1 in mice increases mTORC1 (mTOR complex 1) activation, and this is reversed by treatment with rapamycin, a classical autophagic inducer [72]. PC1 regulates mTORC1 signaling by bringing the soluble tuberous sclerosis complex 2 (TSC2) close to the membrane-bound TSC1, allowing them to form a complex [73]. The TSC1/TSC2 complex works as a GTPase activating protein (GAP) for RHEB (leading to GTP hydrolysis), a small GTPase that is required to activate mTORC1. mTORC1 activation occurs only when RHEB is bound to GTP [74]; therefore, mutation or knockdown of PC1 reduces TSC1/TSC2 complex formation, which causes mTORC1 hyperactivation [73].

In the cpk mouse model of PKD, the kidneys of mutant mice have lower levels of autophagic flux compared to WT animals. This was determined by measuring the levels of LC3-II in WT and cpk mice in the presence of BafA1 [69]. Higher LC3-II and Beclin 1 levels were also observed in rats with PKD (Han:SPRD rats), as evaluated by western blot in kidney tubular epithelial cells [69]. However, in this study, experiments with lysosome blockers were not performed; thus, the possibility that the higher LC3-II levels are caused by an increase in autophagosomes formation over diminished autophagosomes degradation cannot be ruled out.

PC1^−/−^ MEFs do not show an increase in autophagosomes formation (measured by electron microscopy) in the presence of rapamycin [75], suggesting that autophagy is impaired in this cellular model. Also, food restriction, a classic mTORC1-dependent autophagy inducer, does not change LC3 processing in PC1 deficient mice [76]. However, animal models of PKD and PKD patients show an improvement in the symptoms of the disease when treated with rapamycin [67, 68] or food restriction [76]. This suggests that autophagy could be suppressed by mTORC1 in PKD models, but it may not contribute to disease progression (at least in those where PC1 is mutated). Further experiments should be performed to clarify this question. Taken together, however, these studies suggest that mutations of components of the PC in epithelial renal cells lead to the development of PKD, a ciliopathy characterized by two new important cellular features: mTORC1 deregulation and autophagy impairment.

Up to date, PKD is the only ciliopathy that has been linked to autophagy impairment. However, a body of literature indicates that autophagy is dysfunctional in obesity and obesity associated diseases, which, as previously indicated, are a feature of BBS and ALSM1. In the next section, we will review what is known regarding the role of autophagy and the PC in the control of energy homeostasis and body metabolism.

## 6. Energy Homeostasis

### 6.1. Control of Energy Homeostasis and Body Metabolism by Autophagy

Dysregulation of autophagy in different tissues contributes to the development of metabolic disorders such as diabetes and obesity-related diseases (Kim and Lee, 2014; Ryter et al., 2014).

Inhibition of autophagy in the hypothalamus by shRNA-mediated ATG7 knockdown leads to increased body weight due to hyperphagia and decreased energy expenditure, as well as insulin and leptin resistance upon high-fat diet (HFD) feeding, suggesting that hypothalamic autophagy plays a key role in the maintenance of energy homeostasis (Meng et al., 2011; Kim et al., 2013). Meng et al. argue that these effects may depend on I*κ*B kinase *β* (IKK*β*)/nuclear factor- (NF-) *κ*B, as brain-specific IKK*β* knockout abrogates the increase in body weight in mice fed normal chow and HFD [77]. Consistently, the specific deletion of* Atg7* in hypothalamic proopiomelanocortin- (POMC-) expressing neurons increases food intake and reduces energy expenditure, promoting the accumulation of fat mass and body weight [78–80]. In addition to the increase in body weight, deletion of Atg7 in POMC neurons also impairs glucose tolerance [79, 80] and promotes insulin and leptin resistance in mice [78, 79]. Consistently, it has recently been demonstrated that POMC-specific deletion of* Atg12* produces a similar phenotype. Indeed, mice lacking* Atg12* in POMC neurons are obese (due to higher food intake and reduced locomotor activity) and glucose intolerant (Malhotra et al., 2015). Furthermore, these mice exhibit impaired leptin sensitivity and decreased expression of* Pomc* mRNA (Malhotra et al., 2015). Interestingly, mice with a POMC-specific deletion of* Atg5* do not exhibit these phenotypes even if autophagy is impaired (Malhotra et al., 2015).

Interestingly, in contrast to the phenotype where autophagy is prevented in mouse POMC neurons, inhibition of autophagy in orexigenic Agouti-related peptide (AgRP) neurons generates mice with reduced fat mass and decreased hyperphagic response to fasting (Kaushik et al., 2011). These mice have a lean phenotype due to a failure in the upregulation of* AgRP* induced by starvation, which promotes a constitutive increase in POMC and *α*-melanocyte-stimulating hormone (*α*-MSH) expression (Kaushik et al., 2011).

In accordance with the data showing that inhibition of autophagy in the mediobasal hypothalamus and POMC neurons drives obesity and metabolic dysfunction, impaired autophagy is observed in the hypothalamus of rodents challenged with a HFD (Meng et al., 2011) as indicated by the decreased expression of autophagic markers (*Atg5* and* Atg7*), by the lower conversion of LC3-I to LC3-II, and by the increase in p62/SQSTM1 ([77]; Portovedo et al., 2015). Interestingly, hypothalamic modulation of autophagy in response to HFD-induced obesity specifically affects POMC neurons. Indeed, after 20 weeks of 60% HFD feeding, the percentage of POMC neurons with detectable autophagosomes increases more than 10-fold (Thaler et al., 2012). This increase in the number of autophagosomes could be due to an increase in autophagosomes formation or a decrease in autophagosomes degradation in POMC neurons. Future studies are needed to better understand this effect.

The interplay between metabolism and autophagy is not restricted to central nervous system tissues; rather, it operates in many metabolically important tissues. For instance, *β* cell-specific* Atg7*-knockout mice (*Atg*7^Δ*β*  cell^), which lack autophagy in pancreatic *β* cells, are glucose intolerant, hyperglycemic, and hypoinsulinemic because of reduced *β*-cell mass which limits insulin secretion. Indeed, lack of autophagy in these cells promotes their dysfunction and degeneration.* Atg*7^Δ*β*  cell^ mice show inclusion bodies with ubiquitinated material colocalized with p62/SQSTM1, as well as damaged mitochondria and endoplasmic reticulum (ER), which leads to their dysfunction and degeneration (Jung et al., 2008; Ebato et al., 2008). Consistently, adenoviral-mediated depletion of ATG7 in the livers of lean mice leads to insulin resistance, which correlates with ER stress, effects that are significantly improved when ATG7 is reexpressed in hepatocytes [81]. Conversely, the livers of leptin resistant* ob/ob* mice exhibit dysfunctional autophagy as suggested by decreased levels of ATG proteins (LC3, ATG7, and Beclin-1) and increased levels of p62/SQSTM1 [81]. Lack of ATG7 in skeletal muscle (Atg7^Δsm^) enhances glucose clearance and reduces mice body weight due to increased energy expenditure and decreased muscle mass (Masiero et al., 2009). Similar to Atg7^Δsm^ mice, adipose-specific* Atg7* knockout mice are euglycemic, lean, and more sensitive to insulin (Zhang et al., 2009; Singh et al., 2009). Altogether, these studies demonstrate a cell and tissue-specific role of the autophagic process in the regulation of whole-body energy metabolism and in the development of metabolic diseases.

Unfortunately, despite of the fact that is known that the PC controls energy homeostasis at the central and at the peripheral level, in is currently unknown whether these mice models of defective tissue/cell specific-autophagy show altered ciliogenesis/cilia number and/or cilia length. The role the PC in the regulation of energy homeostasis will be discussed in the next section.

### 6.2. Control of Energy Homeostasis by the Primary Cilium at the Hypothalamic Level

Davenport et al. were among the first to characterize the link between PC and obesity. First, the authors demonstrated that the inducible systemic deletion of KIF3A and Tg737 (mouse homologue of IFT88) produces PC loss in adult mice. These mice have increased body weight, fat mass, and food intake compared with their sex- and age-matched controls. Additionally, these mice exhibit nonfasting hyperleptinemia and hyperinsulinemia as well as fasting hyperglycemia. Interestingly, hyperglycemia is prevented in pair-fed mice, suggesting that glucose mishandling is a consequence of hyperphagia-induced obesity [2]. Considering the fundamental role of the hypothalamus in the control of feeding behavior, the authors deleted KIF3A specifically in POMC-expressing neurons in the hypothalamus. In agreement with the previous results, KIF3A knockout mice had higher body weight, fat mass, and food intake compared to their sex- and age-matched controls. Consistently, they also presented increased levels of nonfasting leptin and insulin. Altogether, these results suggest that the depletion of PC in POMC neurons causes hyperphagia-induced obesity [2].

As previously mentioned, BBS is a ciliopathy caused by mutations in at least 19* Bbs* genes, whose protein products are located at the ciliary basal body and axoneme, where they form the BBSome complex [24, 27, 29]. BBS-mouse knockout models (Bbs2^−/−^, Bbs4^−/−^, and Bbs6^−/−^, hereafter named BBS-null mice) show increased body weight associated with hyperphagia, lower locomotor activity, and high plasmatic levels of leptin [33]. Additionally, in a pair-feeding experiment, despite having decreased body weight, BBS-null mice maintained higher visceral fat mass than controls [33], suggesting that, in these mice, the increased adiposity and hyperleptinemia were independent of obesity. While administration of exogenous leptin in WT mice significantly decreased food intake, fat mass, and body weight, in BBS-null mice the effects of leptin were abrogated, suggesting that leptin resistance could contribute to obesity development in BBS-null mice. Additionally, the expression of hypothalamic* Pomc*, but not* Npy* and* Agrp,* was reduced in BBS-null mice [33]. These results suggest that, consistent with the work of Davenport et al., a defect in hypothalamic POMC-expressing neurons contributes to obesity development in this model of PC dysfunction.

Seo et al. also evaluated whether leptin resistance is a cause or a consequence of obesity in BBS-null mice. To test this, mice were caloric restricted (CR), thus preventing obesity and reducing serum leptin levels. Intracerebroventricular (ICV) administration of leptin reduced food intake and body weight in WT mice; however, these effects were abrogated in BBS-null mice, despite normal circulating levels of leptin [82]. These results suggest that ciliary dysfunction in BBS-null mice promotes leptin resistance, which, in turn, causes obesity. To determine the mechanism involved, the authors evaluated the hypothalamic expression of the long isoform of the leptin receptor (LepRb) and the activation of the leptin signaling pathway in BBS-null mice. The mRNA expression of LepRb was similar between WT and BBS-null mice. Nevertheless, the phosphorylation of signal transducer and activator of transcription 3 (STAT3) and the levels of suppressor of cytokine signaling 3 (SOCS3), downstream targets of LepRb, were lower in CR BBS-null mice than WT mice after ICV leptin treatment [82]. These results suggest that the leptin resistance in BBS-null mice is caused by an attenuated response of the LepRb to leptin in the hypothalamus. Consistent with this, Rahmouni et al. also found lower hypothalamic expression of* Pomc*, but not* Npy *and* Agrp*, in knockout mice than controls in basal conditions.

Additionally, Seo et al. demonstrated that LepRb forms a complex with BBS1, which is part of the BBSome complex. Interestingly, depletion of BBS1 or BBS2 caused the retention of LepRb in large vesicles with perinuclear localization, suggesting that BBS proteins are required for the trafficking of LepRb [82]. Consistently, BBS1 M390R, which is the most common mutated form of BBS1 found in patients, shows decreased interaction with LepRb, implying that its trafficking and therefore signaling are altered in BBS patients carrying this mutation. In conclusion, these studies suggest that ciliary BBS proteins are required for trafficking and signaling of the LepRb which, in turn, is necessary for the appropriate hypothalamic response to leptin and correct maintenance of energy balance and body weight [82].

In contrast with the previous studies, data from Berbari et al. indicate that leptin resistance in mice depleted of IFT88 is secondary to obesity. Using mice with an inducible systemic knockout of IFT88 (IFT88Δ/Δ mice), the authors analyzed the response to an intraperitoneal (IP) injection of leptin in preobese, obese, and food-restricted lean mice. IFT88Δ/Δ preobese mice were similar to WT mice in terms of body weight, lean and fat mass, and serum leptin levels. Additionally, IFT88Δ/Δ mice responded to the anorexigenic effect of leptin, an indication that the leptin response remains intact after the depletion of the protein IFT88 and, therefore, the loss of the PC in preobese mice. However, IFT88Δ/Δ mice, after 80 days of ad libitum feeding, became obese and had higher levels of leptin than controls. The anorexigenic effect of leptin was lost in these mice, indicating that leptin resistance is a consequence of obesity in IFT88Δ/Δ mice [34]. However, despite food restriction and body weight loss, IFT88Δ/Δ mice had higher levels of serum leptin than WT mice.

To rule out the possibility that these differences were due to the mouse model that was used, Berbari et al. employed a second model of ciliary dysfunction. They evaluated the leptin response in Bbs4^−/−^ mice before the onset of obesity, observing that preobese Bbs4^−/−^ mice do not show hyperleptinemia or leptin resistance [34], concluding that leptin resistance is a consequence of obesity and hyperleptinemia and not a primary effect associated with PC depletion. These results contrast those obtained by Rahmouni et al. and Seo et al.; in those reports the authors did not analyze mice in a preobese state, which might explain the contradictions.

Additionally, Han et al. described the interplay that exists between hypothalamic PC, leptin, and diet-induced obesity (DIO). In this study, mice were fed with a regular diet or a high-fat and high-sucrose diet for 14 weeks to induce obesity. DIO produced leptin resistance and decreased cilia length, area, and volume specifically in hypothalamic neurons. Similar to DIO mice, the length of hypothalamic PC in both leptin-deficient and leptin receptor-deficient (db/db) mice was also reduced. Interestingly, this effect was reversed by leptin treatment in ob/ob mice, suggesting that the leptin resistance induced by DIO is involved in the regulation of cilia length in hypothalamic neurons [32]. To confirm this hypothesis, the authors exposed N1 hypothalamic neuronal cells to leptin and observed that leptin treatment increased cilia length through the inhibition of PTEN and GSK3*β* proteins. Moreover, in this cell line, LepRb localized at the basal body of the PC [32].

To determine the role of the PC in leptin signaling, Han et al. depleted KIF3A or IFT88 in the mediobasal hypothalamus. This knockdown produced shorter cilia, increased food intake, decreased energy expenditure, and inhibited the anorexigenic effect of ICV injections of leptin, insulin, or glucose [32]. Altogether, these results suggest that DIO produces leptin resistance which, in turns, decreases cilia length in hypothalamic neurons, thus reducing both leptin and insulin sensitivity in these cells.

Additionally, as previously mentioned, leptin treatment elongates the PC without affecting the percentage of ciliated cells in serum-starved N1 hypothalamic neurons, an effect that correlates with a PTEN- and GSK3*β*-dependent increase in the expression of IFT proteins [83]. On the other hand, incubation with leptin or cytochalasin-D increases cilia length in hypothalamic neurons, an effect that is abrogated by treatment with the actin polymerizer phalloidin, evidence that the depolymerization of F-actin is necessary for the stimulatory effects of leptin on cilia length [83].

Another ciliary protein, the retinitis pigmentosa GTPase regulator-interacting protein-1 like (RPGRIP1L), which is localized in the transition zone of the PC, has been implicated in the regulation of body weight. The hypothalamic expression of this gene is reduced by fasting in mice and restored by leptin treatment. In vitro, the knockdown of* Rpgrip1l* in cultured neuronal cells decreases leptin signaling [84] and in vivo, the body weight and the fat mass of Rpgrip1l^+/-^ mice fed with a regular chow diet are higher than those of control mice. Likewise, Rpgrip1l^+/-^ mice fed with chow diet for 18 weeks followed by HFD for a week show higher body weight, fat mass, and lean mass than WT mice [38]. Additionally, Rpgrip1l^+/-^ mice barely respond to leptin injections, which correlates with both a diminished number of ciliated cells and a reduced localization of LepRb at the PC in the arcuate nucleus of the hypothalamus. These data indicate that leptin resistance in Rpgrip1l^+/-^ mice might be associated with a mislocalization of the LepRb [38]. Importantly, reduced numbers of ciliated cells, decreased leptin signaling, and mislocalization of LepRb are also observed in human fibroblasts with hypomorphic mutations in RPGRIP1L derived from subjects with the Joubert syndrome. These effects were reversed by transfection of an intact form of RPGRIP1L [38]. Interestingly, the expression of* Npy* is higher in Rpgrip1l^+/-^ mice than in WTs and its upregulation, caused by fasting, is abrogated. Likewise, the downregulation of* Pomc* expression induced by fasting is inhibited in Rpgrip1l^+/-^ mice. These results support the idea that lack of a ciliary protein, which causes PC depletion, alters the production of neuropeptides that control food intake [33, 38, 82].

Recently, a mutation in the gene encoding for the centrosomal protein 19kDa (CEP19), a protein localized in the centrosome and in the PC, has been associated with morbid obesity in humans and mice [35]. Consistent with other studies that identify a role for the PC in the regulation of food intake and glucose homeostasis [2, 32–34, 38, 82], CEP19 knockout mice are obese, hyperphagic, glucose intolerant, and insulin resistant [35]. Accordingly, mice knocked-out for the gene coding for the protein Ankyrin Repeat Domain 26 (ANKRD26), which functions in protein-protein interactions, which show ciliary defects and mislocalization of the melanin concentrating hormone receptor 1 (MCHR1) and of the somatostatin receptor 3 (SST3R) in the paraventricular nucleus of the hypothalamus, are also hyperphagic and obese [36]. Finally, Jacobs et al. identified a role for the IFT complex A in the regulation of energy metabolism. Using tamoxifen-inducible knockout mice for the protein Tetratricopeptide Repeat Domain 21B (TTC21B, also named IFT139 and THM1), a component of the IFT complex A, the authors demonstrated that loss of this gene increases body weight, body fat percentage, leptin, and insulin and impairs glucose tolerance. Interestingly, while pair-feeding experiments have demonstrated that obesity and glucose intolerance in TTC21B-knockout male mice are caused by hyperphagia, this does not seem to be the case for female mice, where increased body weight is maintained following pair-feeding. [37]. This suggests a dimorphic response to PC dysfunction, which has been observed also in other reports [35, 85]. Additional studies need to be performed to understand the sex-dependent mechanisms that regulate PC signaling. All the cited mouse models of ciliary dysfunction have been summarized in Table 1.

**Figure 4:**
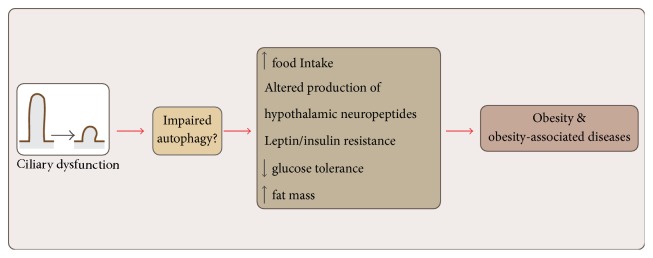
Effects of ciliary dysfunction. Depletion or shortening of PC alters the production of hypothalamic neuropeptides increasing food intake and therefore fat mass. In addition, animals showing ciliary dysfunction are leptin and insulin resistant and intolerant to glucose. In conclusion, depletion or shortening of PC drives obesity and obesity associated diseases. The mechanisms that drive these effects are far from being understood and we propose that this occurs because of an impairment in autophagy. See the main text for additional details.

In conclusion, decrease in PC size or PC ablation in the hypothalamus alters feeding behavior, increases fat mass deposition, changes the expression of neuropeptides, alters leptin response, and reduces glucose tolerance in mice [2, 37, 38] (Figure 4). The cellular and molecular mechanisms involved in the regulation of energy metabolism by the hypothalamic PC remain to be fully elucidated.

### 6.3. Control of Energy Homeostasis by the Primary Cilium at the Peripheral Level

Recently, Gerdes et al., using the Bbs4 mutant mice, evaluated the role of the PC in pancreatic islet function and glucose homeostasis. Four-week-old mice do not show differences when compared to WT mice in islet morphology and serum insulin levels; however, at 7–9 weeks their tolerance to glucose is impaired without affecting body weight, implying that the impaired glucose handling is independent of obesity [86]. Moreover, in whole islets, depletion of two different basal body proteins, OFD1 and BBS4, which lead to PC loss [87] and PC dysfunction, respectively [88], decreases the first phase of insulin secretion, which is one of the earliest anomalies present in patients susceptible to type 2 diabetes [89, 90]. Similarly, MIN6m9 cells depleted of BBS4 showed a decrease in glucose-induced insulin release, which was restored by overexpression of BBS4 [86]. Interestingly, a decrease in the first phase of insulin release after glucose challenge also occurs in *β*-pancreatic cell insulin receptor (IR) knockout mice [91], which points to the interplay between the PC and IR in *β*-pancreatic cells. Moreover, it is known that IR-A localizes at the PC after treatment with insulin in both MIN6m9 and human primary *β*-cells. In addition, the IR needs to be localized at the PC for proper insulin signaling [86]. Finally, diabetic rats (Goto-Kakizaki rats) show a lower number of ciliated *β*-cells than control rats (Wistar). Altogether, these results implicate the PC in *β*-cells as an important player in the development or in the progression of type 2 diabetes.

Consistent with this, a quite recent study showed that BBS proteins are required to maintain IR localization at the cell surface [92]. Indeed, the IR forms a complex with exogenous BBS17, a protein that regulates the trafficking to the ciliary membrane through its interaction with BBS protein complexes [27], and depletion of BBS1, BBS2, BBS6, and BBS12 decreases the amount of IR localized at the plasmatic membrane. Importantly, fibroblasts derived from patients carrying the BBS1 M390R mutation also show reduced surface expression of IR [92]. However, in contrast with Gerdes et al., the authors did not find an overlap between IR and PC, at least in 3T3L1 cells [92]. Nevertheless, the experiment was performed in basal conditions and not after insulin treatment (as Gerdes et al.); thus, it is possible that the IR localizes at the PC, after IR activation.

The role of the PC has been studied also in the adipose tissue. Differentiating preadipocytes, but not proliferating preadipocytes or mature adipocytes, show PC [93, 94]. The size of the PC is highly modified during adipocyte differentiation. Indeed, during the first days of adipocytic differentiation, its size significantly increases, while it progressively decreases hereafter [94]. In addition, Dalbay et al. show that PC elongation is required for adipogenic differentiation [95]. Consistently, Nosavanh and colleagues indicate that the PC is present in brown preadipocytic FVB-C3 cells, a commonly used brown preadipocytic cell line, while it is absent from the same cells when they are proliferating or differentiated. Interestingly, the authors show that the activation of the Hh signaling, whose components localize to the PC, inhibits early brown adipocyte differentiation, when Hh is stimulated [96]. The involvement of the Hh signaling pathway in adipocyte differentiation has been confirmed also in a model of human adipose stem cells where, during adipocyte differentiation, the PC increases its size when Hh is inhibited [94].

Together, these results suggest that the loss of PC favors adipogenesis in the different types of adipose tissue and they identify the PC and PC-dependent signaling as a possible target in the treatment of obesity.

Consistently with these studies, also the downregulation of BBS10 and BBS12 reduces the percentage of ciliated cells while increasing the expression of proadipogenic factors [93]. Likewise, dermal fibroblasts derived from BBS patients show higher triglyceride content and leptin release when compared to healthy fibroblasts [93]. Interestingly, BBS patients, despite being obese, show better tolerance to glucose than BMI-matched non-BBS controls [97]. Furthermore, the subcutaneous adipose tissue of BBS obese patients has higher expression of genes involved in glucose homeostasis* (InsR, Slca4, and Irs)*, proadipogenic genes* (Pparγ, Fas, Acc, Scd2, and Srebp1c),* and anti-inflammatory proteins* (adiponectin, eNOS, and IL10)*, while the expression of proinflammatory markers is diminished* (CD68, iNos, and Tnf-α)* when compared to healthy controls [98]. Importantly, obese Bbs12^−/−^ mice also show lower glucose peaks in glucose tolerance tests and a better hypoglycemic response to insulin when compared to WT mice, consistent with an increased sensitivity to insulin [98]. In addition, after HFD, Bbs12^−/−^ mice have higher body weight, lower fasting glucose levels, and decreased expression of proinflammatory markers along with a significant infiltration of macrophages in the adipose tissue when compared to WT mice [98]. These results indicate that the increased adipogenesis induced by depletion of BBS proteins decreases inflammation, promotes glucose tolerance, and enhances insulin sensitivity despite of the obese state.

## 7. Conclusions

Multiple studies have identified a bidirectional crosstalk between autophagy and the PC [5, 6, 60]. Indeed, cilium-dependent signaling is necessary for autophagosome formation, while autophagy regulates ciliogenesis and cilium length by controlling the degradation of specific ciliary proteins [5, 6]. To date, a limited number of studies have focused on this subject, mainly by evaluating the interplay between autophagy and the PC in condition of nutrient deprivation, without considering other stimuli that modulate the autophagic pathway [5, 6]. In addition, even though it is known that ATG proteins localize at the PC, little is known about their function in this location.

Interestingly, research performed by different groups indicates that both autophagy and the PC are key in the regulation of energy homeostasis, at the central and peripheral levels [2, 3, 77–79, 99, 100]. Lack of autophagy in hypothalamic POMC neurons promotes obesity as well as metabolic dysfunction [77–79]. Consistently, obesity is observed in different models of PC dysfunction caused by hypothalamic hyperphagia and augmented adipocyte differentiation [2, 3, 99]. However, the effect of lack of autophagy in hypothalamic neurons/hypothalamic tissue on ciliogenesis and in the regulation of appetite and energy homeostasis is still unknown.

What is known is that both cilia depleted mice and mice with inhibited autophagy in POMC neurons show impaired response to leptin [78, 80]. Cilia depleted mice have high levels of leptin in plasma caused by hypothalamic leptin resistance and adipocyte-mediated hypersecretion of leptin [2, 101]. POMC-specific* Atg7* knockout mice show increased serum levels of leptin in fasting conditions [78]. However, it is currently unknown if the mechanism that leads to hyperleptinemia in these mice is the same as in cilia depleted mice. Additionally, studies need to better evaluate the effect of autophagic deficiency on ciliogenesis and cilia length in autophagy-deficient mice, as well as how lack of cilia affects the autophagic pathway. Since ciliogenesis and autophagy occur in virtually all the cells of our body, future studies need to be performed in different cell types/tissues to determine if the mechanisms are general or cell type/tissue specific.

As previously mentioned, autophagy dysregulation is a feature of PKD, which, currently, is the only ciliopathy where downregulation of autophagy-related pathways has been studied [67]. Based on the crosstalk between autophagy and ciliogenesis, it would be relevant to determine if autophagy is altered also in other diseases characterized by modified cilia growth. This is of particular relevance if we consider that PKD patients show an improvement in the symptoms of the disease when treated with the autophagic inducer rapamycin, thus suggesting modulation of autophagy might represent a new therapeutic opportunity for treatment of diseases characterized by dysfunctional ciliogenesis.

In conclusion, future research needs to focus on better understanding the interplay that exists between autophagy and ciliogenesis to identify therapeutic targets that, through the modulation of autophagy, might represent effective treatments for different ciliopathies.

## Figures and Tables

**Table 1:** Mice models of ciliary dysfunction and their phenotype.

	Phenotype	Reference
Mediobasal hypothalamus KIF3A and IFT 88 knockdown	↑ food intake	
	↓ energy expenditure	[32]
	↓ anorexigenic effect of ICV injections of leptin/insulin/glucose	

Systemic deletion KIF3A TG737 (IFT88)	↑ body weight	
	↑ food intake	
	Hyperleptinemia	[2]
	Hyperinsulinemia	
	Fasting hyperglycemia	

BBS knockout models Bbs2 −/− Bbs4 −/− Bbs6 −/−	↑ body weight	
	↑ food intake	[33]
	↓ locomotor activity	
	Hyperleptinemia	

IFT88 Δ/Δ mice	↑ body weight	[34]
	Hyperleptinemia	

CEP19 knockout	↑ body weight	
	Hyperphagia	[35]
	↓ glucose tolerance	
	Insulin resistance	

ANK RD26 knockout	↑ Body weight	
	Hyperphagia	[36]
	MCHR1 and SST3R mislocalization in the hypothalamus	

TTC21B knockout	↑ body weight (fat mass)	
	Hyperleptinemia	[37]
	Hyperinsulinemia	
	↓ glucose tolerance	

Rpgrip 1L +/−	↑ body weight (fat mass, lean mass)	
	Leptin resistance	[38]
	Altered production of hypothalamic neuropeptides	

POMC-specific KIF3A KO	↑ body weight	
	↑ food intake	[2]
	Hyperleptinemia	
	Hyperinsulinemia	
